# Response to Brinkmann et al. “Re-assembly of 19th century smallpox vaccine genomes reveals the contemporaneous use of horsepox and horsepox-related viruses in the United States”

**DOI:** 10.1186/s13059-020-02203-z

**Published:** 2020-12-04

**Authors:** Ana T. Duggan, Edward C. Holmes, Hendrik N. Poinar

**Affiliations:** 1 McMaster Ancient DNA Centre, Department of Anthropology, McMaster University, Hamilton, L8S 4L9 Canada; 2 Marie Bashir Institute for Infectious Diseases and Biosecurity, School of Life and Environmental Sciences and School of Medical Sciences, University of Sydney, Sydney, NSW 2006 Australia; 3 M.G. DeGroote Institute for Infectious Disease Research, Department of Biochemistry and Biomedical Sciences, DeGroote School of Medicine, McMaster University, Hamilton, L8S 4L9 Canada

## Abstract

We thank Brinkmann and colleagues for their correspondence and their further investigation into these American Civil War Era vaccination strains. Here, we summarize the difficulties and caveats of work with ancient DNA.

To the Editor:

We are delighted to see that the broad phylogenetic findings and interpretation as presented in our work “The origins and genomic diversity of American Civil War Era smallpox vaccine strains” have been reproduced by Brinkmann and colleagues (Brinkmann A, Souza ARV, Esparza J, Nitsche A, Damaso CR: Re-assembly of 19th-century smallpox vaccine genomes reveals the contemporaneous use of horsepox and horsepox-related viruses in the United States, in preparation) [1]. While the libraries we generated from the vaccination kits likely contain DNA fragments representing the entire genomic content, we question the ability to faithfully reconstruct these termini given the following, well-characterized aspects of ancient DNA (aDNA): (i) The DNA is heavily truncated to median fragments sizes less than 55 bp, (ii) is mixed with the DNA of many different species, and (iii) has damaged nucleotides, features which all complicate mapping and de novo assemblies [2, 3]. These factors exacerbate the fundamental difficulties of re-assembling genomic repetitive elements and regions [4], thereby creating de novo assemblies which are both considerably shorter than full genome length and prone to spurious contigs. We note that the starting material for these libraries were not purified vaccine materials, but rather scabrous material and old tin boxes. The resulting extracts produced complex metagenomic libraries, further increasing the likelihood of generating of chimeric contigs and chimeric genome reassemblies. Furthermore, our libraries were biased by multiple sequencing strategies—including both shotgun and targeted enrichment—particularly important when considering novel insertions such as those proposed by Brinkmann et al. Assembly algorithms and software specifically designed for the challenges of aDNA are scarce, as such researchers typically err on the side of caution. Methodologies involving full or partial manual assemblies are avoided as they are necessarily subjective and therefore difficult to reproduce. On that final point, we note that while the increased length and diversity of terminal repetitive regions identified by Brinkmann et al. could be very interesting, within the sphere of aDNA research increased diversity and unexpected length variation are also red flags for chimeric sequence reconstruction.
